# Linking biochemical and individual-level effects of chlorpyrifos, triphenyl phosphate, and bisphenol A on sea urchin (*Paracentrotus lividus*) larvae

**DOI:** 10.1007/s11356-022-19099-w

**Published:** 2022-02-14

**Authors:** Juan Bellas, Diego Rial, Juliana Valdés, Leticia Vidal-Liñán, Juan I. Bertucci, Soledad Muniategui, Víctor M. León, Juan A. Campillo

**Affiliations:** 1 Centro Oceanográfico de Vigo, Instituto Español de Oceanografía (IEO, CSIC), Subida a Radio Faro 50, 36390 Vigo, Spain; 2 Centro Oceanográfico de Murcia, Instituto Español de Oceanografía (IEO, CSIC), Varadero 1, San Pedro del Pinatar, 30740 Murcia, Spain; 3 Grupo Química Analítica Aplicada (QANAP), Instituto Universitario de Medio Ambiente (IUMA), Centro de Investigaciones Científicas Avanzadas (CICA), Departamento de Química Analítica, Facultade de Ciencias, Universidade da Coruña, Campus de A Coruña, 15071 A Coruña, Spain

**Keywords:** Sea urchin, Embryo-larval bioassay, Biomarkers, Acetylcholinesterase, Antioxidant enzymes, Glutathione S-transferase

## Abstract

The effects of three relevant organic pollutants: chlorpyrifos (CPF), a widely used insecticide, triphenyl phosphate (TPHP), employed as flame retardant and as plastic additive, and bisphenol A (BPA), used primarily as plastic additive, on sea urchin (*Paracentrotus lividus*) larvae, were investigated. Experiments consisted of exposing sea urchin fertilized eggs throughout their development to the 4-arm pluteus larval stage. The antioxidant enzymes glutathione reductase (GR) and catalase (CAT), the phase II detoxification enzyme glutathione S-transferase (GST), and the neurotransmitter catabolism enzyme acetylcholinesterase (AChE) were assessed in combination with responses at the individual level (larval growth). CPF was the most toxic compound with 10 and 50% effective concentrations (EC_10_ and EC_50_) values of 60 and 279 μg/l (0.17 and 0.80 μM), followed by TPHP with EC_10_ and EC_50_ values of 224 and 1213 μg/l (0.68 and 3.7 μM), and by BPA with EC_10_ and EC_50_ values of 885 and 1549 μg/l (3.9 and 6.8 μM). The toxicity of the three compounds was attributed to oxidative stress, to the modulation of the AChE response, and/or to the reduction of the detoxification efficacy. Increasing trends in CAT activity were observed for BPA and, to a lower extent, for CPF. GR activity showed a bell-shaped response in larvae exposed to CPF, whereas BPA caused an increasing trend in GR. GST also displayed a bell-shaped response to CPF exposure and a decreasing trend was observed for TPHP. An inhibition pattern in AChE activity was observed at increasing BPA concentrations. A potential role of the GST in the metabolism of CPF was proposed, but not for TPHP or BPA, and a significant increase of AChE activity associated with oxidative stress was observed in TPHP-exposed larvae. Among the biochemical responses, the GR activity was found to be a reliable biomarker of exposure for sea urchin early-life stages, providing a first sign of damage. These results show that the integration of responses at the biochemical level with fitness-related responses (e.g., growth) may help to improve knowledge about the impact of toxic substances on marine ecosystems.

**Supplementary Information:**

The online version contains supplementary material available at 10.1007/s11356-022-19099-w.

## Introduction

A variety of biological measures can potentially be used to evaluate the risk of damage caused by environmental pollutants to marine ecosystems, as long as they fulfill three fundamental conditions that allow obtaining relevant results at the ecosystem level: (i) to be easily standardizable, rapid, and cost-effective; (ii) to be sufficiently sensitive to the toxic effect of pollutants; and (iii) to have implications on the biological fitness of the individual (e.g., mortality, growth, reproduction, feeding rates) (Stebbing et al. 1980; Rand et al. 1995; Calow 1998). The responses to be measured can take place at different levels of biological organization. Responses at the lowest levels of organization are usually sensitive and specific, and may be good indicators of pollutant exposure (Viarengo et al. 2007; Lam 2009; Beiras 2018). The validity of responses at cellular, molecular, and biochemical level, so-called biomarkers, has been well-established in the assessment of marine pollution (McCarthy and Shugart 1990; Viarengo et al. 2007; Beiras 2018). The measurement of biomarkers could serve as a diagnostic tool to understand the effect of pollutants on the health of organisms and to provide an “early-warning” signal of pollution damage on ecosystems (McCarthy and Shugart 1990; Viarengo et al. 2007; Lam 2009; Martinez-Haro et al. 2015; Beiras 2018). However, the application of biomarkers in laboratory and field studies, including large-scale monitoring programs, has also revealed their main weakness which is the limited knowledge of their ecological significance regarding the structure and function of the population or ecosystem (Bellas et al. 2014; Campillo et al. 2019), since the mechanistic link with individual- or population-level responses is usually not straightforward (Maltby et al. 2001). In this respect, it has been recommended that enzymatic biomarkers of exposure (e.g., the antioxidant enzymes glutathione reductase and catalase or the phase II detoxification enzyme glutathione S-transferase), representing compensatory responses of the organism (Campillo et al. 2013; Regoli and Giuliani 2014; Quetglas-Llabrés et al. 2020), should be used in combination with biomarkers of effect (e.g., indicators of damage such as the neurotransmitter catabolism enzyme acetylcholinesterase) (Bocquené and Galgani 1998; Campillo et al. 2013; Vidal-Liñán and Bellas 2013), that may help to understand the potential consequences of these alterations on the health status of the organisms (Viarengo et al. 2007).

According to recent environmental legislation (e.g., the Water Framework Directive, 2000/60/EC or the Marine Strategy Framework Directive, 2008/56/EC), that has shifted towards a holistic view, where the ecosystem is used as the fundamental unit of environmental management (Bellas et al. 2014), the way forward for the application of biological responses in marine pollution monitoring is the integration of responses at the molecular and biochemical levels with responses at higher levels of biological organization (e.g., Adverse Outcome Pathway concept, Ankley et al. 2010; Martinez-Haro et al. 2015). In this way, a compromise between the sensitivity and mechanistic explanation of the early signals of pollution damage and the ecological relevance of fitness-related responses would be achieved, which may allow taking preventive measures before the ecological damage occurs (Clements 2000; Maltby et al. 2001).

The life stages of marine organisms show differences in their sensitivity to pollutants and, if the success of a species depends on its performance as it passes through successive phases of development, it is logical to use the most sensitive life stages to assess environmental quality (Stebbing et al. 1980). It has been shown that the early developmental stages of marine invertebrates are more sensitive to toxicants than adults (e.g., Marin et al. 1991) and are therefore particularly suitable for assessing marine pollution. In fact, since Wilson’s pioneer work in the 1950s, liquid-phase bioassays with embryos and larvae of marine invertebrates have achieved an advanced stage of development (Kobayashi 1995; His et al. 1999) and have become widely used routine tools by international agencies such as the US Environmental Protection Agency (Dinnel et al. 1989) or the International Council for the Exploration of the Sea (Beiras et al. 2012) to assess the water quality of coastal ecosystems. These bioassays can be appropriate systems to integrate responses at the biochemical level with fitness-related responses, as previously advocated (Clements 2000; Maltby et al. 2001; Martinez-Haro et al. 2015), contributing to a better understanding of the impact of toxicants in marine ecosystems.

In this study, we aim to determine the effects of three environmentally relevant organic pollutants: chlorpyrifos (CPF), a neurotoxic organophosphate compound commonly employed as insecticide (John and Shaike 2015); triphenyl phosphate (TPHP), an organophosphate compound widely used as flame retardant and plasticizer (Wei et al. 2015); and bisphenol A (BPA), an endocrine-disrupting phenol derivative used primarily as plasticizer (López-Pacheco et al. 2019), on larvae of the sea urchin *Paracentrotus lividus*. With that aim, four biochemical biomarkers widely employed in marine pollution studies were selected: the antioxidant enzymes glutathione reductase (GR) and catalase (CAT), the phase II detoxification enzyme glutathione S-transferase (GST), and the neurotransmitter catabolism enzyme acetylcholinesterase (AChE) (Bocquené and Galgani 1998; Vidal-Liñán et al. 2010; Campillo et al. 2013; Regoli and Giuliani 2014; Quetglas-Llabrés et al. 2020). CAT and GR are part of the antioxidant defense system that protect cells against the harmful effects of excess reactive oxygen species (ROS) resulting from exposure to pollutants (Regoli and Giuliani 2014). CAT catalyzes the breakdown of hydrogen peroxide into water and oxygen and GR catalyzes the reduction of glutathione disulfide (GSSG) to glutathione (GSH), maintaining the GSSG/GSH balance essential for cellular homeostasis (Kirk 1969). GSTs are conjugation enzymes involved in the detoxification of organic compounds, which also play a role in protection against oxidative stress (Sheehan et al. 2001). AChE regulates the transmission of the nerve impulse by hydrolysis of the neurotransmitter acetylcholine. AChE activity is inhibited by certain toxicants, altering the nerve impulse and leading to severe physiological effects (Bocquené and Galgani 1990).

This study also aimed to integrate the biochemical effects of the selected pollutants with effects at the individual level (larval growth) in order to understand the toxicants mode of action and to establish the ecological meaning of the biochemical responses, which currently represents one of the main challenges in ecotoxicology.

## Materials and methods

### Biological material

Mature *P. lividus* were collected in a pristine site located at the outer part of the Ría de Vigo (Galicia, NW Spain). Animals were transported to the laboratory in a portable icebox and maintained in aquaria with running natural seawater for 1 week until the experiments. Handling conditions of the adult stock were 13.38 ± 0.85 °C temperature, 34.41 ± 0.77 ppt salinity, and 7.66 ± 0.17 mg/l O_2_ (mean ± std). Sea urchins were fed with the green algae *Ulva lactuca*. Gametes were obtained by dissection of adults, and their maturity (egg sphericity and sperm mobility) was checked with a microscope according to Beiras et al. (2012). The eggs were transferred to a 100-ml graduated cylinder containing seawater, ca. 10 μl of the sperm was added, and the mixture was gently shaken to facilitate the fertilization. Aliquots of 20 μl were taken to count the total number of eggs and fertilized eggs in a Sedgewick-Rafter counting chamber.

### Experimental solutions

Selected biocides were analytical grade CPF (O,O-diethyl O-3,5,6-trichloropyridin-2-yl phosphorothioate); BPA (4,4′-(propane-2,2-diyl)diphenol); and TPHP ((C_6_H_5_)_3_PO_4_), acquired from Sigma-Aldrich (Merck Life Science S.L.U., María de Molina 40, 28006 Madrid, Spain). Stock solutions were freshly prepared by dissolving the product in dimethyl sulfoxide (DMSO). The final concentration of DMSO never exceeded 0.01% (v/v), which had been previously found to be non-toxic to sea urchin embryos and larvae (Bellas et al. 2005).

The experimental concentrations, obtained by serial dilution of the stock solution in filtered seawater (FSW, 1 μm filtered UV-irradiated) with oceanic characteristics, were below the water saturation levels of the tested compounds.

### Experimental procedure

Exposure experiments carried out to define the toxicity of the three compounds consisted of delivering 350 fertilized eggs (i.e., those with a fertilization membrane) into glass vials with airtight Teflon-lined screw caps containing 20 ml of the experimental solutions. Experimental concentrations were based on previous work of the research group and on data from literature (e.g., Bellas et al. 2005; Lin 2009; Tato et al. 2018). The concentrations assayed were 2, 5, 10, 50, 70, 100, 200, 400, 800, and 1000 μg/l for CPF; 200, 400, 600, 800, 1000, 1200, 1500, 2000, 3000, and 5000 μg/l for BPA; and 1, 50, 100, 200, 500, 1000, and 1900 μg/l for TPHP. The vials were incubated in darkness for 48 h at 20 °C (until larvae reached the four-arm pluteus stage). Four replicates per treatment, including controls (FSW) and DMSO controls (0.01% v/v), were assayed for each experiment. After the incubation period, sea urchin larvae were preserved by adding a few drops of 40% buffered formalin and the mean larval growth (*n* = 35) was recorded. Larval growth was defined as the maximum dimension in the first 35 individuals per vial (including embryos), subtracting the average of the diameter of the fertilized eggs (90.49 ± 4.53 μm) (Figure S1). Length of individuals was recorded using an inverted microscope (Axiovert 40 CFL, Carl Zeiss Microscopy GmbH, Jena, Germany) coupled to a DFK 42BUC03 digital camera (The Imaging Source Europe GmbH, Bremen, Germany) and Micro-Manager image analysis software version 1.4.17 (https://micro-manager.org, Vale Lab, University of California San Francisco, USA). The EC_10_ and EC_50_ were defined as the toxicant concentrations causing 10% and 50% reduction in larval growth. Control embryogenesis success was always above 90%. Physico-chemical conditions of the experiments were (mean ± SD) 35.10 ± 0.64 psu salinity, 7.58 ± 0.01 mg/l O_2_, and 8.23 ± 0.01 pH.

Exposure experiments for biomarker analysis were conducted by delivering approximately 80,000 fertilized eggs (> 97% fertilization rate) into glass beakers containing 2 l of the experimental solutions, reaching a density of 40 embryos/ml. The concentrations assayed were 10, 35, 70, 140, and 280 μg/l for CPF; 100, 200, 400, 800, and 1200 μg/l for BPA; and 1, 50, 100, 250, and 1000 μg/l for TPHP. Four replicates per treatment were performed. Exposure beakers were gently aerated with 0.22 μm filtered air and were allowed to equilibrate for 1 h, before introducing the embryos. The beakers were incubated in darkness at 20 °C for 48 h. After the incubation period, samples of ca. 100 sea urchin larvae per replicate were preserved by adding a few drops of 40% buffered formalin, and the mean larval growth (*n* = 35) was recorded as mentioned above. The remaining larvae were filtered through 50-μm nylon mesh, collected in Eppendorf tubes, and processed for subsequent biomarker analysis.

All glassware was acid-washed (HNO_3_, 10% vol) and rinsed with acetone and distilled water before the experiments.

### Biochemical analyses

Larval pools were homogenized at 4 °C in 1:3 wet weight/buffer volume ratio in 100 mM phosphate buffer, pH 7.5 containing 1 mM DTT and 1 mM EDTA, using a sonifier (Hielscher UP200S) by 2 × 15 s sonication steps (0.5 cycle, 40 amplitude). Samples were then centrifuged at 9000 × g during 10 min at 4 °C. The supernatant (S9 fraction) was separated in aliquots and stocked at a − 80 °C freezer for posterior measurement of enzymatic activities.

Biochemical measurements were carried out on Nicolet Evolution 300 spectrophotometer (Thermo Fisher Scientific Inc., USA) and Spectrafluor plus microplate reader (Tecan Group Ltd., Switzerland). All the enzymatic determinations were carried out at 25 °C. AChE was determined by a modification of the Ellman method adapted for microplate (Bocquené and Galgani 1998). AChE activity was measured in the presence of 2.6 mM acetylthiocholine and 0.5 mM 5,5-dithiobis-2-dinitrobenzoic acid, and the increase of absorbance was measured at 412 nm. GST activity was measured according to Habig et al. (1974) using chlorodinitrobenzene as substrate. The formation of S-2,4-dinitrophenyl glutathione conjugate was monitored following its absorbance at 340 nm (extinction coefficient, *ε* = 9.6 mM/cm). CAT activity was measured at 240 nm (*ε* = − 0.04 mM/cm) in an assay mixture that contained 50 mM K-phosphate buffer pH 7.0 and 50 mM H_2_O_2_ (Claiborne 1985). GR activity was measured at 340 nm (*ε* = − 6.22 mM/cm) in an assay mixture that contained 100 mM K-phosphate buffer pH 7.0, 1 mM GSSG, and 0.06 mM NADPH (Ramos-Martínez et al. 1983). Assays were run at least in duplicate. In all cases, results were expressed in relation to the protein concentration of each subcellular fraction determined according to Lowry et al. (1951).

### Chemical analyses

Water samples were taken at 0 h to check the initial concentrations of each toxicant. CPF and TPPH were analyzed in seawater samples by stir bar sorptive extraction coupled to gas chromatography-mass spectrometry (SBSE/GC/MS), following the method proposed by Rial et al. (2017) and optimized in this study adding TPPH analysis. Briefly, the procedure consisted of an SBSE stage with 100 g/l NaCl using commercial polydimethylsiloxane stir bars (Gerstel, Mulheim a/d Ruhr, Germany) shaken at 700 rpm for 22 h, after which analytes were thermo-desorbed from the stir and analyzed by GC/MS in full-scan mode. CPF-D10 was used as internal standard. The limits of quantification (LOQ) were established for a signal to noise ratio of 10, from a standard solution of 10 ng/l. The LOQ were 0.4 ng/l for CPF and 0.6 ng/l for TPPH, showing good reproducibility and repeatability.

BPA was analyzed in seawater samples by dispersive liquid-liquid microextraction followed by high-performance liquid chromatographic determination coupled to tandem mass spectrometry (DLLME-LC-MS/MS), using an Agilent® 1200 series HPLC and Triple Quadrupole (QqQ) 3200 (Applied Byosystems®), with electrospray ionization (ESI) source (see details in Salgueiro-González et al. 2012). Samples were analyzed in duplicate (DER% < 5). The LOQ was 0.02 μg/l.

### Statistical analyses

Data were analyzed for normality and homogeneity of variances using the Shapiro-Wilk test and the Levene test, respectively. Data that failed to pass homogeneity tests were log-transformed and re-tested. Data were then analyzed by one-way ANOVA, followed by the Student-Newman-Keuls post hoc test at a significance level of *p* < 0.05. Tests were performed using Infostat (Di Rienzo et al. 2013). Pearson correlation analysis and principal component analysis (PCA) were conducted to establish relationships between biological responses and to select the most relevant biomarkers. Correlation analysis was performed using R (Mei and Yu 2018) and PCA was performed using the SPSS statistical package. A threshold factor loading of 0.5 was chosen to represent a good association between variables and components. Multiple linear regression was performed using the *lm* function in R. Larval growth was chosen as the independent variable and concentration and biomarker responses (CAT, GR, GST, and AChE) as explanatory variables.

The cumulative function of the Weibull distribution was used for describing the reduction in growth of sea urchin larvae exposed to pollutants (Rial et al. 2010):

1 $$R=K-K\left\{1-\exp\left[-in2\left(\frac Cm\right)^a\right]\right\}$$

where *R* is the response, *K* is the maximum value of the response, *C* is the concentration, *m* is the dose corresponding to the semi-maximum response, and *a* is a shape parameter.

A Gaussian model was used to describe those patterns of biomarker response with a bell shape (Motulsky 2021):

2 $$R=\mathrm{Base}+\mathrm{Amplitude}\times e^{\left(-{0.5}^x\left(\left(C-\mathrm{Mean}\right)/\mathrm{SD}\right)^\wedge2\right)}$$

where *R* is the response, Base is the minimum value of the ordinates, Amplitude is the height of the distribution in *Y* units, Mean is the abscissa at the center of the distribution, and SD is a measure of the width of the distribution.

## Results

### Analysis of contaminants in seawater samples

Analysis of seawater samples revealed that initial CPF, BPA, and TPHP concentrations ranged between 74 and 105%, 94 and 99%, and 80 and 85% of the nominal concentrations, respectively (Table 1). Nominal concentrations were used for data analysis and presentation of the results.

**Table 1:** Nominal and measured concentrations (μg/l) of chlorpyrifos, triphenyl phosphate, and bisphenol A, at the beginning of the experiments (*t*_0_)

	Nominal concentration (μg/l)	Measured concentration (μg/l)
Chlorpyrifos	0	< 0.020
	1	0.74
	10	7.6
	100	105
Triphenyl phosphate	0	< 0.020
	1	0.82
	100	79.9
	1000	854
Bisphenol A	0	< 0.020
	100	94
	400	397
	1200	1126

### Larval growth and biomarker analysis

**Fig. 1:**
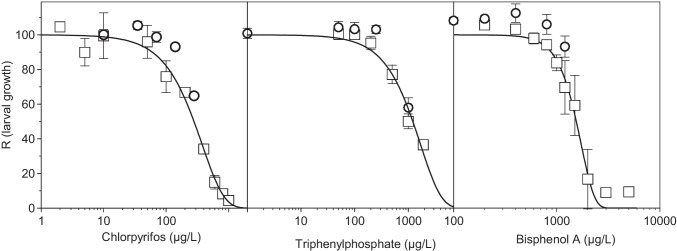
Inhibition of sea urchin larval growth by chlorpyrifos (left), triphenyl phosphate (center), and bisphenol A (right). Square: exposure experiments carried out to define the toxicity of each substance. Circle: exposure experiments for biomarker analysis. Lines represent the fittings of experimental data to Eq. 1 for the square. Bars represent standard errors

CPF, BPA, and TPHP affected the embryonic development of the sea urchin *P. lividus* within the experimental concentration range in a dose-dependent manner (Figure 1, Figure S1). According to the sigmoidal dose-response model (Eq. 1) used to describe the reduction in growth of sea urchin larvae exposed to these compounds, a strong relationship was found between the concentration of CPF (*R*^2^ = 0.985, *p* < 0.0001), BPA (*R*^2^ = 0.985, *p* < 0.0001), and TPHP (*R*^2^ = 0.964, *p* < 0.0001) and the larval growth inhibition (Table 2). CPF showed the highest toxicity, with EC_10_ and EC_50_ values of 60 and 279 μg/l (0.17 and 0.80 μM), respectively (Table 2). TPHP showed lower toxicity than CPF yielding EC_10_ and EC_50_ values of 224 and 1213 μg/l (0.68 and 3.7 μM), respectively. BPA was the less toxic compound, with EC_10_ and EC_50_ values of 885 and 1549 μg/l (3.9 and 6.8 μM), respectively.

**Table 2:** Summary of the parametric estimates and confidence intervals (*α* = 0.05) obtained by fitting Eq. 1 to the growth of sea urchin larva exposed to chlorpyrifos, triphenyl phosphate, and bisphenol A. Statistical values of adjusted coefficient of multiple determination (adj. *R*^2^) and *p* values from Fisher’s *F* test (*α* = 0.05) are also summarized

Parameter	Chlorpyrifos	Triphenyl phosphate	Bisphenol A
*K*	1	1	1
*m*/EC_50_ (μg/l)	278.8 ± 41.0	1212.9 ± 251.1	1549.0 ± 109.3
*a*	1.2 ± 0.3	1.1 ± 0.4	3.4 ± 1.0
EC_10_ (μg/l)	60.0 ± 24.2	223.5 ± 136.4	884.6 ± 157.1
adj. *R*^2^	0.985	0.964	0.985
*p* value	0.000	0.000	0.000

Regarding the experiments carried out for biomarker analysis, a significant decrease in larval growth was observed in groups treated with 140 and 280 μg CPF/l (Figure 1). The exposure of embryos to 1000 μg/l of TPHP caused a > 40% growth inhibition. The treatment with BPA caused a slight significant increase in larval growth at 400 μg/l and a significant decrease at the highest concentration tested (1200 μg/l).

**Fig. 2:**
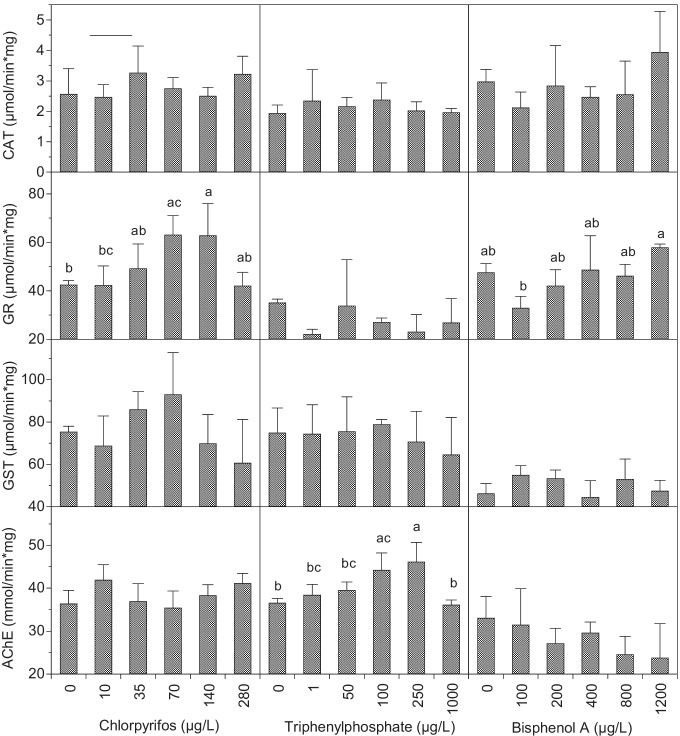
Catalase (CAT), glutathione reductase (GR), glutathione S-transferase (GST), and acetylcholinesterase (AChE) activities in sea urchin larvae exposed to chlorpyrifos (left), triphenyl phosphate (center), and bisphenol A (right). “0”: DMSO controls (< 0.01 % v/v). Data were analyzed by one-way ANOVA, followed by post hoc Student-Newman-Keuls test at a significance level of *p* < 0.05. Bars represent standard errors

No significant alteration of CAT enzymatic activity was detected after exposure to CPF, TPHP, or BPA (Figure 2). However, increasing trends in CAT activity were observed for BPA and, to a lower extent, for CPF.

**Fig. 3:**
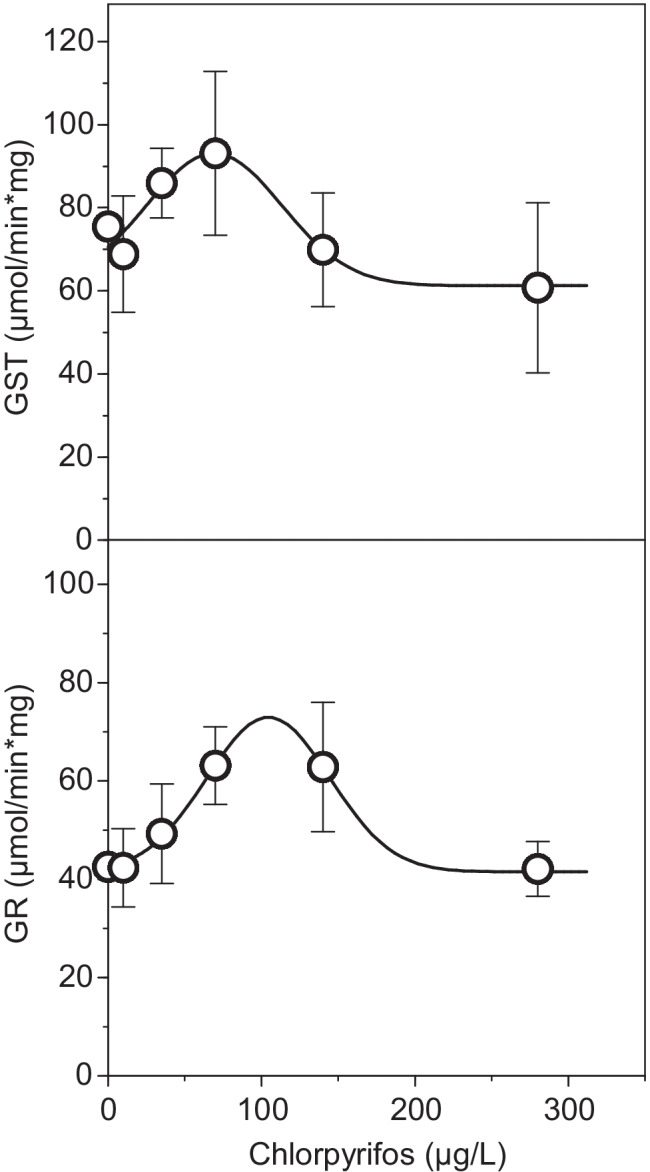
Glutathione S-transferase (GST, up) and glutathione reductase (GR, down) activities in sea urchin larva exposed to chlorpyrifos. Lines represent the predictions of the model described by Eq. 2. Bars represent standard errors

A significant induction of GR activity was observed at increasing CPF concentrations up to 140 μg/l, followed by a decrease to control levels at the highest concentration tested (280 μg/l) (Figure 2). This response pattern is characterized by a bell-shaped curve and fits well with a Gaussian model (Figure 3, Table S1, *R*^2^ = 0.798, *p* < 0.01). No significant variation of GR enzymatic activity was detected after exposure to TPHP. BPA caused an increasing trend in GR activity (Figure 2, *R*^2^ = 0.548, *p* = 0.09), although no significant alterations were detected with respect to the control.

GST also displayed a bell-shaped response to CPF exposure (Figures 2 and 3); although, in this case, the fit to the Gaussian model was not significant (Table S1, *R*^2^ = 0.618, *p* = 0.147). The highest GST activity values were observed at 70 μg/l and a decrease was observed at the two highest CPF concentrations (140 and 280 μg/l). No significant differences in GST activity were found at any of the TPHP or BPA tested concentrations with respect to controls, although a significant decreasing trend was observed for TPHP (Figure 2, *R*^2^ = − 0.792, *p* < 0.05).

No significant alteration of the AChE activity was registered after CPF or BPA exposure at any of the tested concentrations, but an inhibition trend was observed at increasing BPA concentrations (Figure 2, *R*^2^ = 0.790, *p* < 0.05). TPHP exposure caused a significant increase of the AChE activity at 100 and 250 μg/l, and the activity value returned to the control levels at the highest tested concentration (1000 μg/l) (Figure 2).

### Correlations between molecular biomarkers

**Fig. 4:**
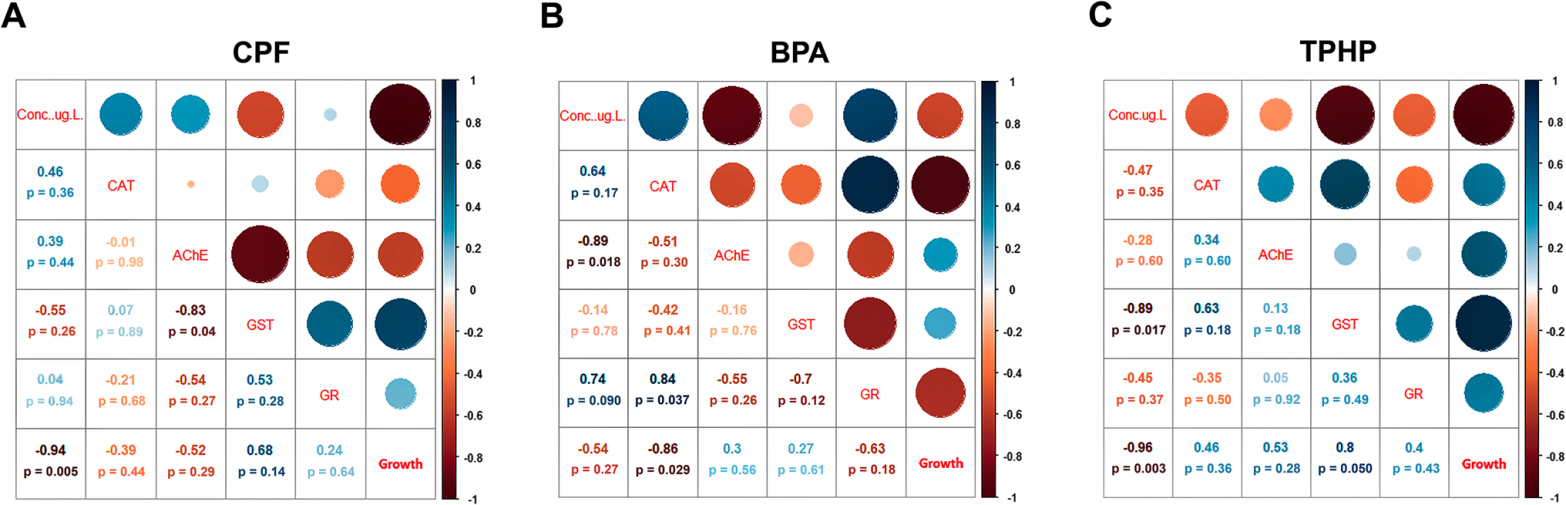
Pearson correlation coefficients between enzymatic activities, larval growth, and CPF (**A**), BPA (**B**), and TPHP (**C**) concentrations

Pearson correlation analysis evidenced that the relationships between biomarkers were toxicant-specific. The strongest correlations for CPF exposure were detected between GST and AChE (*R*^2^ = − 0.832), and between GST and GR (*R*^2^ = − 0.532), but only the inverse correlation with AChE was significant (*p* < 0.05; Figure 4A). Regarding BPA exposure, the strongest correlations were found between GR and CAT (*R*^2^ = 0.839) and between GR and GST (*R*^2^ = − 0.697), being statistically significant the positive correlation between GR and CAT (*p* < 0.05; Figure 4B). A positive, although not statistically significant, correlation between GST and CAT (Figure 4C, *R*^2^ = 0.630) was found for TPHP.

### Correlations between molecular biomarkers and larval growth

A satisfactory description of the sea urchin larval growth was obtained from the explanatory variables of the multiple linear regression model for CPF (adj. R^2^ = 0.904, p < 0.01) and TPHP (adj. R^2^ = 0.891, p < 0.001); not so for BPA (adj. R^2^ = 0.274, p = 0.112) (Table S2). In the case of CPF, the explanatory variables Concentration and GR showed a statistically significant relationship with larval growth. For TPHP, the most relevant explanatory variables were Concentration and AChE. A correlation of larval growth with CAT activity was detected for BPA though the general fit of the model was poor.

### Principal component analysis

**Fig. 5:**
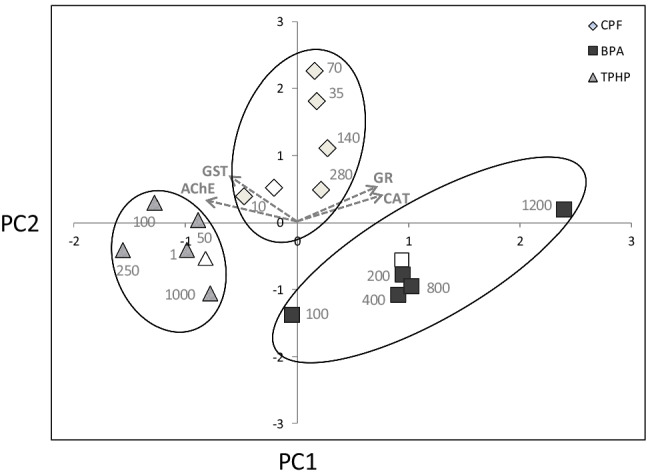
Two-dimensional plot of the principal component analysis for the biomarker responses (gray arrows), showing the different CPF (square), TPHP (diamond), and BPA (circle) treatments. Open symbols represent controls

The two principal components of the PCA explained 83.5% of the total variation (Figure 5). PC1 (56.0% of total variance) was defined by high loadings of the four enzymatic activities: AChE (− 0.850), CAT (0.771), GR (0.736), and GST (− 0.618). Thus, larvae with higher oxidative stress, according to CAT and GR levels, would have a lower activity of the phase II detoxification enzyme GST and a decrease in levels of the neurotoxicity biomarker AChE. PC2 (27.4% of the total variance) was defined mainly by GST (0.723) and to a lower extent by GR (0.549). Sea urchin larvae exposed to CPF, TPHP, and BPA were clearly discriminated by the PCA. CPF-exposed larvae were associated to the positive part of PC2. The value of PC2 increased at increasing CPF concentrations, up to 70 μg/l, and decreased at the two highest CPF concentrations (140 and 280 μg/l), according to the variation of GST and GR activities, that increase at lower CPF concentrations and decrease at higher CPF concentrations. TPHP-exposed larvae were mainly distributed in the negative part of both PC1 and PC2. These larvae showed a similar pattern than those exposed to CPF, with an increase of PC2 values at increasing TPHP concentrations up to 100 μg/l and a subsequent decrease at the two highest concentrations (250 and 1000 μg/l), linked to a decrease in GST levels. BPA-exposed larvae were located in the positive part of PC1 and in the negative part of PC2. In this case, the response to the toxicant is linked to both PC1 and PC2, and is defined by the increase in GR and CAT and the decrease in AChE levels.

## Discussion

The toxic effects of three selected organic substances, the organophosphate insecticide CPF, the organophosphate flame retardant and plasticizer TPHP, and the endocrine-disrupting phenol derivative BPA, on early stages of development of the sea urchin *P. lividus*, were studied. The occurrence of these pollutants has been reported not only in coastal areas but also at certain distance from pollution sources (León et al. 2020; Zhong et al. 2017). Seawater concentrations of 0.20, 0.0008, and 0.48 μg/l have been recorded for CPF, TPHP, and BPA (Campillo et al. 2013; Zhong et al. 2017; Salgueiro-González et al. 2019). CPF, TPHP, and BPA provoked alterations in the sea urchin larval growth after 48 h of incubation at the tested concentrations. The EC_10_ and EC_50_ values for larval growth obtained in the present study agree with previous research carried out with sea urchin embryos for CPF (Bellas et al. 2005; Buono et al. 2012) and BPA (Kiyomoto et al. 2006; Özlem and Hatice 2008; Tato et al. 2018), reporting effects at similar concentrations than those reported here. However, no information is available on the toxicity of TPHP on sea urchin embryos and limited toxicological information has been obtained on early stages of aquatic organisms, mainly on zebrafish (*Danio rerio*), reporting effects within the range of 40–29,600 μg/l (geometric mean: 650 μg/l) (e.g., Isales et al. 2015; Jarema et al. 2015; Achenbach et al. 2020). These effect concentrations are, in general, higher than concentrations detected in seawater for these substances (usually < 0.5 μg/l) (Campillo et al. 2013; Liu et al. 2018; Salgueiro-González et al. 2019), although higher concentrations can be present close to main pollution sources or, in the case of plastic additives, close to high plastic debris accumulation.

Regarding the biomarker responses, sea urchin larvae exposed to each toxicant were discriminated by the PCA, probably due to the different mechanisms of toxicity of these substances during embryonic and larval development. A typical bell-shaped response was observed in GR activity after CPF exposure. The GR catalyzes the reduction of GSSG to the sulfhydryl form, GSH (Kirk 1969). GSH plays an essential role in protecting against oxidative stress and in the maintenance of the reducing environment of the cell, acting as a key cellular antioxidant that is also involved in phase II biotransformation of xenobiotics (Kehrer et al. 2010). The bell-shaped response pattern observed here for GR is common in enzymes whose activity is induced by exposure to environmental pollutants, when the exposure occurs above a certain threshold (Viarengo et al. 2007). The findings presented here suggest that CPF exposure produces oxidative stress, increasing the levels of ROS that at low and medium concentrations results in the induction of the enzyme synthesis, causing an increase in the enzymatic activity in order to control the oxidative stress. This is in agreement with other studies that reported the induction of oxidative stress after exposure to organic chemicals, including CPF, in aquatic and terrestrial organisms (Kavitha and Rao 2008; Wu et al. 2011; Patetsini et al. 2013; Negro et al. 2019; Banaee et al. 2020). In fact, an important role of ROS production in the toxicity mechanism of CPF has been reported in fish (Botté et al. 2012). Conversely, higher CPF concentrations lead to a decrease of the GR activity, which may be due to the alteration of the oxidative stress balance, the direct inhibition of the enzyme caused by the toxicant, and/or the increase in the catabolic rate (Viarengo et al. 2007). As a result, the GR would not be able to reduce the produced GSSG to GSH and modulate the effects of CPF (Regoli and Giuliani 2014).

GSTs are phase II detoxification enzymes that catalyze the conjugation of GSH to a range of organic compounds, preventing them from reacting with target molecules in the cell and making them more water-soluble and more easily excreted (Sheehan et al. 2001; Balogh and Atkins 2011). There is abundant literature on the increase of the GST activity in response to a wide range of xenobiotics, although a high interspecific variability has been reported (Hayes et al. 2005). The GST activity of CPF-exposed sea urchin larvae shows a similar bell-shaped response pattern than GR, decreasing at the highest CPF concentrations, and therefore suggesting a potential role of the GST pathway in the metabolism of CPF, as had been previously indicated (Fujioka and Casida 2007). This finding is supported by the distribution of CPF-exposed sea urchin larvae along the positive part of PC2, defined mainly by the variation of GST and GR activities. However, other studies did not find evidence of the involvement of GST in the detoxification of CPF in marine organisms such as mussels or fish, and a species-dependent response of the GST activity to CPF has been suggested (Botté et al. 2012; Perić and Burić 2019). Moreover, the increase in GST may also be associated with the metabolism and elimination of oxidative stress products, such as the reduction of lipid peroxides or the regeneration S-thiolated proteins (Singhal et al. 1994; Sheehan et al. 2001; Pham et al. 2002). On the other hand, since GST and GR are linked through the GSH recycling system, the GST decrease observed at high CPF concentrations is probably related to the decrease in the content of available GSH due to GR dysfunction (Regoli and Giuliani 2014), which is also reflected in the decreasing values of CPF-exposed larvae along the PC2 axis at the highest CPF concentrations. Inhibition of GST activity has also been reported in fish species exposed to CPF (Kavitha and Rao 2008) and CPF mixtures (Wang et al. 2009). This decrease in antioxidant defenses and phase II response ultimately affects the normal growth of the sea urchin larvae (as indicated by the multiple linear regression model) which is significantly impaired at the highest CPF concentrations.

The CAT activity did not show the same response pattern as GST or GR in CPF-exposed sea urchin embryos. ROS metabolization appears to take place mainly by glutathione peroxidases (GPx), as indicated by the initial increase in GR activity to regenerate GSH. GPx use the reduced form of GSH to decompose peroxides yielding GSSG which is regenerated through an active GR redox cycle (Regoli and Giuliani 2014). The increase in GR activity under stress conditions, linked to that of GPx, has been observed in different organisms (González-Fernández et al. 2015; Haque et al. 2019). Although CAT is predominant in H_2_O_2_ detoxification, catalyzing the decomposition of H_2_O_2_ into water and oxygen, GPx is the first antioxidant defense mechanism to be activated since it has more affinity than CAT to H_2_O_2_ (Vidal-Liñán et al. 2010). In this line, CAT would only increase when oxidative stress is very high, explaining the different response patterns between CAT and GR.

Limited information is available about the mode of action of TPHP. Developmental neurotoxicity, alteration of neurotransmitters, and gene and protein expression in the central nervous system have been observed in zebrafish larvae (Shi et al. 2018). In the present study, no effect of TPHP exposure was observed on CAT and GR activities, despite oxidative stress has previously been reported after exposure to other organophosphates in vivo and in vitro, in fish (e.g., the zebrafish, Du et al. 2019) and marine invertebrates (e.g., the mussel, *Mytilus galloprovincialis*, Meng et al. 2019). GST activity remained unchanged at low TPHP concentrations, but a decreasing trend was observed at higher concentrations, which resulted in a reduction of the detoxification efficacy, as shown by the decreasing values of TPHP-exposed larvae along the PC2 axis at the highest TPHP concentrations. The decreased levels of this phase II biotransformation enzyme resulted in a > 40% inhibition of larval growth at the highest TPHP concentration.

BPA acts as an endocrine disruptor since its mode of action in biological systems is through steroidal hormone mimicking (Gorini et al. 2020). As other endocrine disruptors, BPA also induces oxidative stress causing damage in several tissues, especially during early development (Dutta and Paul 2018, 2019). BPA exposure caused increasing trends of GR and CAT activities in sea urchin embryos, as shown by the PCA distribution in the positive part of PC1. No effect of BPA on the GST activity was observed, though, suggesting that GST is not involved in the metabolism of BPA in sea urchin embryos. The positive correlation between GR and CAT which, in turn, is negatively correlated with larval growth supports the idea that BPA is generating oxidative stress in sea urchin embryos that leads to a delay in their growth at the highest concentration. Likewise, Goto et al. (1992) found that the progress of mouse embryonic development in vitro was delayed by oxidative stress (protein-thiol oxidation). However, a significant increase in larval growth was observed at 400 μg BPA/l, which might be related to the activation and interplay of several detoxification processes (e.g., defense mechanisms against ROS). Usually, at low or medium concentrations, the detoxification processes occur to modulate the effects of xenobiotics and maintain the normal function of the organism. This is evidenced here by the normal growth of the sea urchin larvae, which may even increase as a secondary effect, at the lowest toxicant concentrations. In this line, Bošnjak et al. (2014) evidenced the role of the multidrug efflux transporter P-glycoprotein (P-gp/ABCB1) in the protection of *P. lividus* embryos and larvae against BPA. However, beyond a certain point, further increases in the toxicant concentration may overwhelm the detoxification system, generating the accumulation of toxic metabolites and leading to adverse effects (Depledge et al. 1993; Jung et al. 2001). In fact, Arslan and Parlak (2016) reported genotoxic damage (increased micronuclei frequencies) in *P. lividus* embryos exposed to BPA.

The inhibition of AChE (and the associated neurotoxicity) by organophosphate pesticides is a well-studied response (Kavitha and Rao 2008; Wu et al. 2011). In this work, no significant inhibition of AChE activity was observed after CPF and TPHP exposure. As has been previously demonstrated, early sea urchin embryos develop a functional cholinergic response system, including the expression of the essential receptors and the signaling cascades (Buznikov et al. 1996; Buznikov et al. 2001; Qiao et al. 2003). Neurotransmitters such as acetylcholine are present in these embryos, playing different roles in regulatory processes during ontogenesis (gametogenesis, fertilization, cleavage divisions, or early cell interactions), even before the development of the nervous system (Buznikov et al. 1996). AChE is also found in early sea urchin embryos but its activity is not high, increasing sharply during gastrulation (Buznikov 1984; Buznikov et al. 1996). The release of neurotransmitters at early developmental stages takes place by direct release, without enzymatic inactivation, whereas during and before gastrulation, the enzymatic excretion becomes increasingly important (Buznikov 1984). This may explain the low sensitivity of the AChE activity to CPF and TPHP observed in this study. In this regard, in other sea urchin species (*Hemicentrotus pulcherrimus*), the AChE activity at 24 h was insignificant compared to that at 48 h after hatching and increased four times from 36 to 48 h (Zhang et al. 2017). When *H. pulcherrimus* larvae were exposed to the organophosphate pesticide monocrotophos, significant AChE inhibition occurred only after 48 h, being even higher than controls after 36 h (Zhang et al. 2017). On the other hand, in contrast to what is expected, the present study reports a significant increase of AChE activity in TPHP-exposed sea urchin larvae. Several studies have suggested that elevated AChE activity levels are associated with oxidative stress (Melo et al. 2003; Rosemberg et al. 2010; Santos et al. 2012). Moreover, Reutgard and Furuhagen (2016) linked the increase of AChE and oxidative stress with the frequency of malformed embryos and arrested embryo development in the benthic amphipod *Monoporeia affinis*. Cholinesterases play an important role in regulating cell-to-cell interactions and other relevant biological events such as those occurring during the embryonic development (Falugi and Aluigi 2012). As suggested by these authors, understanding the regulation mechanisms of these events can help to establish the impact of neurotoxic substances on the environment. These AChE functions may provide an explanation to the association between the AChE activity and the decrease in larval growth indicated by the multiple linear regression model for TPHP.

The mechanism of action of organophosphates, mainly based on the inhibition of the AChE activity is, in the case of CPF, mediated by its metabolite chlorpyrifos oxon (Buznikov et al. 2001). Thus, an explanation for the lack of AChE inhibition under CPF exposure in sea urchin embryos could be related to the lack, unlike in fish, of a CPF metabolism pathway capable of generating the oxon derivative that causes the AChE inhibition by reacting with its active center. However, non-cholinergic neurotoxic effects of CPF have also been reported in sea urchin embryos, even at lower concentrations than AChE inhibition (Buznikov et al. 2001; Buznikov et al. 2007). Interestingly, a significant inhibition pattern in the AChE activity was observed with increasing BPA concentrations, which suggests a neurotoxic effect of BPA, as has previously been reported for freshwater species (Chen et al. 2017; Liu et al. 2019). Therefore, considering that AChE activity increases markedly at later developmental stages, these results suggest that BPA could significantly generate neurotoxicity in *P. lividus* development.

Summing up, the present work reports a decrease in sea urchin larval growth after exposure of newly fertilized embryos to CPF, BPA, and TPHP for 48 h that may have implications on the fitness of the population. This alteration of the larval growth would be attributed to oxidative damage, to the modulation of the AChE response, and/or to the reduction of the detoxification efficacy, during embryonic and larval development. Sea urchin embryos exposed to CPF, BPA, and TPHP showed different patterns of response, probably due to the different ability of these chemicals to produce ROS and activate defense mechanisms. These biological responses have been widely used as pollutant biomarkers in adult marine organisms to show adverse effects before they are irreversible. Changes in these biochemical parameters in early life stages of development may be relevant for the assessment of the toxicity mechanisms of pollutants and also have ecological significance, since they appear to be related to growth; so they may contribute to understanding the impact of toxic substances on marine ecosystems. Among the studied oxidative stress responses, the GR activity has been found to be a reliable biomarker of exposure to chemical agents, providing a first sign of damage caused by pollutants on sea urchin early-life stages. A potential role of the GST in the metabolism of CPF is proposed, but not in the metabolism of TPHP or BPA. Regarding BPA, an inhibition pattern of the AChE activity is shown that could be more pronounced at later stages of development. On the other hand, increasing levels of AChE activity were found at the lower concentrations of TPHP, followed by a decrease to the control values at higher concentrations, when larval growth was affected. The interaction between AChE and antioxidant enzymes in response to organophosphate compounds is suggested, which should be interesting to address in future works including chronic exposures of sea urchin larvae.

## Supplementary Information

Below is the link to the electronic supplementary material.

Supplementary file1 (DOCX 234 kb)

## Data Availability

The datasets used and/or analyzed during the current study are available from the corresponding author on reasonable request.

## References

[CR1] Achenbach JC, Leggiadro C, Sperker SA, Woodland C, Ellis LD (2020). Comparison of the zebrafish embryo toxicity assay and the general and behavioral embryo toxicity assay as new approach methods for chemical screening. Toxics.

[CR2] Ankley GT, Bennett RS, Erickson RJ, Hoff DJ, Hornung MW, Johnson RD, Mount DR, Nichols JW, Russom CL, Schmieder PK, Serrano JA, Tietge JE, Villeneuve DL (2010). Adverse outcome pathways: a conceptual framework to support ecotoxicology research and risk assessment. Environ Toxicol Chem.

[CR3] Arslan OC, Parlak H (2016). Sea urchin micronucleus assay for determination of genotoxic effects of bisphenol A. Fresenius Environ Bull.

[CR4] Balogh LM, Atkins WM (2011). Interactions of glutathione transferases with 4-hydroxynonenal. Drug Metab Rev.

[CR5] Banaee M, Akhlaghi M, Soltanian S, Sureda A, Gholamhosseini A, Rakhshaninejad M (2020). Combined effects of exposure to sub-lethal concentration of the insecticide chlorpyrifos and the herbicide glyphosate on the biochemical changes in the freshwater crayfish *Pontastacus leptodactylus*. Ecotoxicology.

[CR6] Beiras R (2018). Marine pollution: sources, fate and effects of pollutants in coastal ecosystems.

[CR7] Beiras R, Durán I, Bellas J (2012) Biological effects of contaminants: *Paracentrotus lividus* sea urchin embryo test with marine sediment elutriates. ICES Techniques in Marine. Environ Sci 51 13 pp

[CR8] Bellas J, Beiras R, Marino-Balsa JC, Fernández N (2005). Toxicity of organic compounds to marine invertebrate embryos and larvae: a comparison between the sea urchin embryogenesis bioassay and alternative test species. Ecotoxicology.

[CR9] Bellas J, Albentosa M, Vidal-Liñán L, Besada V, Franco MA, Fumega J, González-Quijano A, Viñas L, Beiras R (2014). Combined use of chemical, biochemical and physiological variables in mussels for the assessment of marine pollution along the N-NW Spanish coast. Mar Environ Res.

[CR10] Bocquené G, Galgani F (1990). Characterization and assay conditions for use of AChE activity from several marine species in pollution monitoring. Mar Environ Res.

[CR11] Bocquené G, Galgani F (1998) Biological effects of contaminants: cholinesterases inhibition by organophosphate and carbamate compounds. ICES Techniques in Marine. Environ Sci 22 13 pp

[CR12] Bošnjak I, Borra M, Iamunno F, Benvenuto G, Ujević I, Bušelić I, Roje-Busatto R, Mladineo I (2014). Effect of bisphenol A on P-glycoprotein-mediated efflux and ultrastructure of the sea urchin embryo. Aquat Toxicol.

[CR13] Botté ES, Jerry DR, King SC, Smith-Keune C, Negri AP (2012). Effects of chlorpyrifos on cholinesterase activity and stress markers in the tropical reef fish *Acanthochromis polyacanthus*. Mar Pollut Bull.

[CR14] Buono S, Manzo S, Maria G, Sansone G (2012). Toxic effects of pentachlorophenol, azinphos-methyl and chlorpyrifos on the development of *Paracentrotus lividus* embryos. Ecotoxicology.

[CR15] Buznikov GA (1984). The action of neurotransmitters and related substances on early embryogenesis. Pharmacol Ther.

[CR16] Buznikov GA, Shmukler YB, Lauder JM (1996). From oocyte to neuron: do neurotransmitters function in the same way throughout development?. Cell Mol Neurobiol.

[CR17] Buznikov GA, Nikitina LA, Bezuglov VV, Lauder JM, Padilla S, Slotkin TA (2001). An invertebrate model of the developmental neurotoxicity of insecticides: effects of chlorpyrifos and dieldrin in sea urchin embryos and larvae. Environ Health Perspect.

[CR18] Buznikov GA, Nikitina LA, Rakić LM, Milošević I, Bezuglov VV, Lauder JM, Slotkin TA (2007). The sea urchin embryo, an invertebrate model for mammalian developmental neurotoxicity, reveals multiple neurotransmitter mechanisms for effects of chlorpyrifos: therapeutic interventions and a comparison with the monoamine depleter, reserpine. Brain Res Bull.

[CR19] Calow P (1998). Ecological risk assessment: risk for what? How do we decide?. Ecotoxicol Environ Saf.

[CR20] Campillo JA, Albentosa M, Valdés NJ, Moreno-González R, León VM (2013). Impact assessment of agricultural inputs into a Mediterranean coastal lagoon (Mar Menor, SE Spain) on transplanted clams (*Ruditapes decussatus*) by biochemical and physiological responses. Aquat Toxicol.

[CR21] Campillo JA, Sevilla A, González-Fernández C, Bellas J, Bernal C, Cánovas M, Albentosa M (2019). Metabolomic responses of mussel *Mytilus galloprovincialis* to fluoranthene exposure under different nutritive conditions. Mar Environ Res.

[CR22] Chen Q, Yin D, Jia Y, Schiwy S, Legradi J, Yang S, Hollert H (2017). Enhanced uptake of BPA in the presence of nanoplastics can lead to neurotoxic effects in adult zebra fish. Sci Total Environ.

[CR23] Claiborne A, Greenwald RA (1985). Catalase activity. Handbook of methods for oxygen radical research.

[CR24] Clements WH (2000). Integrating effects of contaminants across levels of biological organization: an overview. J Aquat Ecosyst Stress Recover.

[CR25] Depledge MH, Amaral-Mendes JJ, Daniel BRSH, Halbrook RS, Kloepper-Sams P, Moore MN, Peakall DB, Peakall DG, Shugart LR (1993). The conceptual basis of the biomarker approach. Biomarkers.

[CR26] Di Rienzo J, Casanoves F, Balzarini M, Gonzalez L, Tablada M, Robledo C (2013) Infostat - Sofware estadístico. Universidad Nacional de Córdoba, Argentina. http://www.infostat.com.ar/

[CR27] Dinnel PA, Link JM, Stober QJ, Letourneau MW, Roberts WE (1989). Comparative sensitivity of sea urchin sperm bioassays to metals and pesticides. Arch Environ Contam Toxicol.

[CR28] Du J, Li H, Xu S, Zhou Q, Jin M, Tang J (2019). A review of organophosphorus flame retardants (OPFRs): occurrence, bioaccumulation, toxicity, and organism exposure. Environ Sci Pollut Res.

[CR29] Dutta M, Paul G (2018). Bisphenol A dose- and time-dependently induces oxidative stress in rat liver mitochondria ex vivo. Asian J Pharm Clin Res.

[CR30] Dutta M, Paul G (2019). Gallic acid protects rat liver mitochondria ex vivo from bisphenol A induced oxidative stress mediated damages. Toxicol Rep.

[CR31] Falugi C, Aluigi MG (2012). Early appearance and possible functions of non-neuromuscular cholinesterase activities. Front Mol Neurosci.

[CR32] Fujioka K, Casida JE (2007). Glutathione S-transferase conjugation of organophosphorus pesticides yields S-phospho-, S-aryl-, and S-alkylglutathione derivatives. Chem Res Toxicol.

[CR33] González-Fernández C, Albentosa M, Campillo JA, Viñas L, Romero D, Franco A, Bellas J (2015). Effect of nutritive status on *Mytilus galloprovincialis* pollution biomarkers: implications for large-scale monitoring programs. Aquat Toxicol.

[CR34] Gorini F, Bustaffa E, Coi A, Iervasi G, Bianchi F (2020). Bisphenols as environmental triggers of thyroid dysfunction: clues and evidence. Int J Environ Res Public Health.

[CR35] Goto Y, Noda Y, Narimoto K, Umaoka Y, Mori T (1992). Oxidative stress on mouse embryo development in vitro. Free Radic Biol Med.

[CR36] Habig WH, Pabst MJ, Jakoby WB (1974). Glutathione S-tranferase. The first enzymatic step in mercapturic acid formation. J Biol Chem.

[CR37] Haque MN, Eom H-J, Nam S-E, Shin YK, Rhee J-S (2019). Chlorothalonil induces oxidative stress and reduces enzymatic activities of Na+/K+-ATPase and acetylcholinesterase in gill tissues of marine bivalves. PLoS One.

[CR38] Hayes JD, Flanagan JU, Jowsey IR (2005). Glutathione transferases. Annu Rev Pharmacol Toxicol.

[CR39] His E, Beiras R, Seaman MNL (1999). The assessment of marine pollution bioassays with bivalve embryos and larvae. Adv Mar Biol.

[CR40] Isales GM, Hipszer RA, Raftery TD, Chen A, Stapleton HM, Volz DC (2015). Triphenyl phosphate-induced developmental toxicity in zebrafish: potential role of the retinoic acid receptor. Aquat Toxicol.

[CR41] Jarema KA, Hunter DL, Shaffer RM, Behl M, Padilla S (2015). Acute and developmental behavioral effects of flame retardants and related chemicals in zebrafish. Neurotoxicol Teratol.

[CR42] John EM, Shaike JM (2015). Chlorpyrifos: pollution and remediation. Environ Chem Lett.

[CR43] Jung DKJ, Klaus T, Fent K (2001). Cytochrome P450 induction by nitrated polycyclic aromatic hydrocarbons, azaarenes, and binary mixtures in fish hepatoma cell line PLHC-1. Environ Toxicol Chem.

[CR44] Kavitha P, Rao JV (2008). Toxic effects of chlorpyrifos on antioxidant enzymes and target enzyme acetylcholinesterase interaction in mosquito fish, *Gambusia affinis*. Environ Toxicol Pharmacol.

[CR45] Kehrer JP, Robertson JD, Smith CV, McQueen CA (2010). Free radicals and reactive oxygen species. Comprehensive toxicology.

[CR46] Kirk JE, Kirk JE (1969). Oxidoreductases. Enzymes of the arterial wall.

[CR47] Kiyomoto M, Kikuchi A, Unuma T, Yokota Y (2006). Effects of ethynylestradiol and bisphenol A on the development of sea urchin embryos and juveniles. Mar Biol.

[CR48] Kobayashi N, Cheremisinoff PN (1995). Bioassay data for marine pollution using echinoderms. Encyclopedia of environmental control technology.

[CR49] Lam PK (2009). Use of biomarkers in environmental monitoring. Ocean Coast Manag.

[CR50] León VM, Viñas L, Concha-Graña E, Fernández-González V, Salgueiro-González N, Moscoso-Pérez C, Muniategui-Lorenzo S, Campillo JA (2020). Identification of contaminants of emerging concern with potential environmental risk in Spanish continental shelf sediments. Sci Total Environ.

[CR51] Lin K (2009). Joint acute toxicity of tributyl phosphate and triphenyl phosphate to *Daphnia magna*. Environ Chem Lett.

[CR52] Liu L, Tang J, Zhong J, Zhen X, Pan X, Tian C (2018). Spatial distribution and seasonal variation of four current-use pesticides (CUPs) in air and surface water of the Bohai Sea, China. Sci Total Environ.

[CR53] Liu Y, Yan Z, Zhang L, Deng Z, Yuan J, Zhang S, Chen J, Guo R (2019). Food up-take and reproduction performance of *Daphnia magna* under the exposure of bisphenols. Ecotoxicol Environ Saf.

[CR54] López-Pacheco IY, Silva-Núñez A, Salinas-Salazar C, Arévalo-Gallegos A, Lizarazo-Holguin LA, Barceló D, Iqbal HMN, Parra-Saldívar R (2019). Anthropogenic contaminants of high concern: existence in water resources and their adverse effects. Sci Total Environ.

[CR55] Lowry OH, Rosenbrough NJ, Farr AL, Randall RJ (1951). Protein measurement with the folin phenol reagent. J Biol Chem.

[CR56] Maltby L, Kedwards TJ, Forbes VE, Grasman K, Kammenga JE, Munns WR, Ringwood AH, Weis JS, Wood SN, Baird DJ, Burton GA (2001). Linking individual-level responses and population-level consequences. Ecological variability: separating natural from anthropogenic causes of ecosystem impairment.

[CR57] Marin MG, Bressan M, Brunetti R (1991). Effects of linear alkylbenzene sulphonate (LAS) on two marine benthic organisms. Aquat Toxicol.

[CR58] Martinez-Haro M, Beiras R, Bellas J, Capela R, Coelho JP, Lopes I, Moreira-Santos M, Reis-Henriques AM, Ribeiro R, Santos MM, Marques JC (2015). A review on the ecological quality status assessment in aquatic systems using community based indicators and ecotoxicological tools: what might be the added value of their combination?. Ecol Indic.

[CR59] McCarthy JF, Shugart LR (1990). Biomarkers of environmental contamination.

[CR60] Mei W, Yu G (2018) basicTrendline: add trendline and confidence interval of basic regression models to plot. R package

[CR61] Melo JB, Agostinho P, Oliveira CR (2003). Involvement of oxidative stress in the enhancement of acetylcholinesterase activity induced by amyloid beta-peptide. Neurosci Res.

[CR62] Meng X, Li F, Wang X, Liu J, Ji C, Wu H (2019). Combinatorial immune and stress response, cytoskeleton and signal transduction effects of graphene and triphenyl phosphate (TPP) in mussel *Mytilus galloprovincialis*. J Hazard Mater.

[CR63] Motulsky HJ (2021) GraphPad Curve Fitting Guide. http://www.graphpad.com/guides/prism/7/curve-fitting/index.htm. Accessed 12 July 2021

[CR64] Negro CL, Iturburu FG, Mendieta J, Menone ML, Collins P (2019). Are oxidative stress biomarkers sensitive to environmental concentrations of chlorpyrifos exposed to the freshwater crab, *Zilchiopsis collastinensis* (Decapoda; Trichodactylidae)?. Bull Environ Contam Toxicol.

[CR65] Özlem CA, Hatice P (2008). Effects of bisphenol A on the embryonic development of sea urchin (*Paracentrotus lividus*). Environ Toxicol.

[CR66] Patetsini E, Dimitriadis VK, Kaloyianni M (2013). Biomarkers in marine mussels, *Mytilus galloprovincialis*, exposed to environmentally relevant levels of the pesticides, chlorpyrifos and penoxsulam. Aquat Toxicol.

[CR67] Perić L, Burić P (2019). The effect of copper and chlorpyrifos co-exposure on biomarkers in the marine mussel *Mytilus galloprovincialis*. Chemosphere.

[CR68] Pham RT, Gardner JL, Gallagher EP (2002). Conjugation of 4-hydroxynonenal by largemouth bass (*Micropterus salmoides*) glutathione S-transferases. Mar Environ Res.

[CR69] Qiao D, Nikitina LA, Buznikov GA, Lauder JM, Seidler FJ, Slotkin TA (2003). The sea urchin embryo as a model for mammalian developmental neurotoxicity: ontogenesis of the high-affinity choline transporter and its role in cholinergic trophic activity. Environ Health Perspect.

[CR70] Quetglas-Llabrés MM, Tejada S, Capó X, Langley E, Sureda A, Box A (2020). Antioxidant response of the sea urchin *Paracentrotus lividus* to pollution and the invasive algae *Lophocladia lallemandii*. Chemosphere.

[CR71] Ramos-Martínez JI, Rodrigez-Bartolomé T, Vázquez-Pernas R (1983). Purification and properties of glutathione reductase from hepatopancreas of *Mytilus edulis* L. Comp Biochem Physiol B Comp Biochem.

[CR72] Rand GM, Wells PG, McCarty LS, Rand GM (1995). Introduction to aquatic toxicology. Fundamentals of aquatic toxicology: effects, environmental fate, and risk assessment.

[CR73] Regoli F, Giuliani ME (2014). Oxidative pathways of chemical toxicity and oxidative stress biomarkers in marine organisms. Mar Environ Res.

[CR74] Reutgard M, Furuhagen S (2016). Linking sub-cellular biomarkers to embryo aberrations in the benthic amphipod Monoporeia affinis. Aquat Toxicol.

[CR75] Rial D, Beiras R, Vázquez JA, Murado MA (2010). Acute toxicity of a shoreline cleaner, Cytosol, mixed with oil and ecological risk assessment of its use on the Galician coast. Arch Environ Contam Toxicol.

[CR76] Rial D, León VM, Bellas J (2017). Integrative assessment of coastal marine pollution in Bay of Santander and Galician Rías. J Sea Res.

[CR77] Rosemberg DB, da Rocha RF, Rico EP, Zanotto-Filho ALFEU, Dias RD, Bogo MR, Bonan CD, Moreira JCF, Klamt F, Souza DO (2010). Taurine prevents enhancement of acetylcholinesterase activity induced by acute ethanol exposure and decreases the level of markers of oxidative stress in zebrafish brain. Neuroscience.

[CR78] Salgueiro-González N, Concha-Graña E, Turnes-Carou I, Muniategui-Lorenzo S, López-Mahía P, Prada-Rodríguez D (2012). Determination of alkylphenols and bisphenol A in seawater samples by dispersive liquid–liquid microextraction and liquid chromatography tandem mass spectrometry for compliance with environmental quality standards (Directive 2008/105/EC). J Chromatogr A.

[CR79] Salgueiro-González N, Campillo JA, Viñas L, Beiras R, López-Mahía P, Muniategui-Lorenzo S (2019). Occurrence of selected endocrine disrupting compounds in Iberian coastal areas and assessment of the environmental risk. Environ Pollut.

[CR80] Santos D, Milatovic D, Andrade V, Batoreu MC, Aschner M, Dos Santos AM (2012). The inhibitory effect of manganese on acetylcholinesterase activity enhances oxidative stress and neuroinflammation in the rat brain. Toxicology.

[CR81] Sheehan D, Meade G, Foley VM, Dowd CA (2001). Structure, function and evolution of glutathione transferase for classification of non-mammalian members of an ancient superfamily. Biochem J.

[CR82] Shi Q, Wang M, Shi F, Yang L, Guo Y, Feng C, Liu J, Zhou B (2018). Developmental neurotoxicity of triphenyl phosphate in zebrafish larvae. Aquat Toxicol.

[CR83] Singhal SS, Zimniak P, Sharma R, Srivastava SK, Awasthi S, Awasthi YC (1994). A novel glutathione S-transferase isozyme similar to GST 8-8 of rat and mGSTA4-4 (GST 5.7) of mouse is selectively expressed in human tissues. Biochim Biophys Acta (BBA)-Protein Struct Mol Enzymol.

[CR84] Stebbing ARD, Akesson B, Calabrese A, Gentile JH, Jensen A, Lloyd R (1980). The role of bioassays in marine pollution monitoring. Rapports Procès-Verbaux Réunions Comm Int Explor Sci Mer Méditerranée.

[CR85] Tato T, Salgueiro-González N, León VM, González S, Beiras R (2018). Ecotoxicological evaluation of the risk posed by bisphenol A, triclosan, and 4-nonylphenol in coastal waters using early life stages of marine organisms (*Isochrysis galbana*, *Mytilus galloprovincialis*, *Paracentrotus lividus*, and *Acartia clausi*). Environ Pollut.

[CR86] Viarengo A, Lowe D, Bolognesi C, Fabbri E, Koehler A (2007). The use of biomarkers in biomonitoring: a 2-tier approach assessing the level of pollutant-induced stress syndrome in sentinel organisms. Comp Biochem Physiol C Toxicol Pharmacol.

[CR87] Vidal-Liñán L, Bellas J (2013). Practical procedures for selected biomarkers in mussels, *Mytilus galloprovincialis* e implications for marine pollution monitoring. Sci Total Environ.

[CR88] Vidal-Liñán L, Bellas J, Campillo JA, Beiras R (2010). Integrated use of antioxidant enzymes in mussels, *Mytilus galloprovincialis*, for monitoring pollution in highly productive coastal areas of Galicia (NW Spain). Chemosphere.

[CR89] Wang C, Lu G, Cui J, Wang P (2009). Sublethal effects of pesticide mixtures on selected biomarkers of *Carassius auratus*. Environ Toxicol Pharmacol.

[CR90] Wei GL, Li DQ, Zhuo MN, Liao YS, Xie ZY, Guo TL, Li JJ, Zhang SY, Liang ZQ (2015). Organophosphorus flame retardants and plasticizers: sources, occurrence, toxicity and human exposure. Environ Pollut.

[CR91] Wu H, Zhang R, Liu J, Guo Y, Ma E (2011). Effects of malathion and chlorpyrifos on acetylcholinesterase and antioxidant defense system in *Oxya chinensis* (Thunberg) (Orthoptera: Acrididae). Chemosphere.

[CR92] Zhang X, Li S, Wang C, Tian H, Wang W, Ru S (2017). Effects of monocrotophos pesticide on cholinergic and dopaminergic neurotransmitter systems during early development in the sea urchin *Hemicentrotus pulcherrimus*. Toxicol Appl Pharmacol.

[CR93] Zhong M, Tanga J, Mi L, Li F, Wanga L, Huang G, Wu H (2017). Occurrence and spatial distribution of organophosphorus flame retardants and plasticizers in the Bohai and Yellow Seas, China. Mar Pollut Bull.

