# Supplementary material for: Linking biochemical and individual-level effects of chlorpyrifos, triphenyl phosphate, and bisphenol A on sea urchin (Paracentrotus lividus) larvae
Source: Environ Sci Pollut Res Int. 2022 Feb 14;29(30):46174–87. doi: 10.1007/s11356-022-19099-w (PMC9209388; doi:10.1007/s11356-022-19099-w)
Supplement: Supplementary file 1 — Supplementary file1 (DOCX 234 kb) [file 11356_2022_19099_MOESM1_ESM.docx]

# Supplementary material


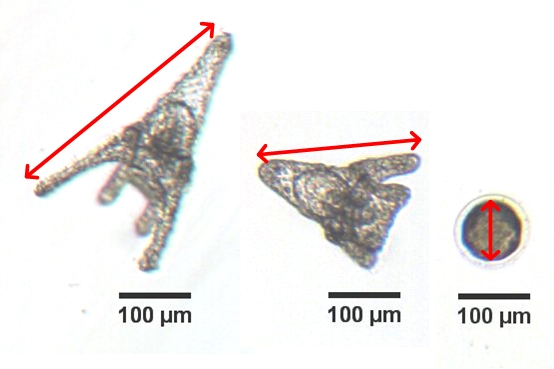


B

A


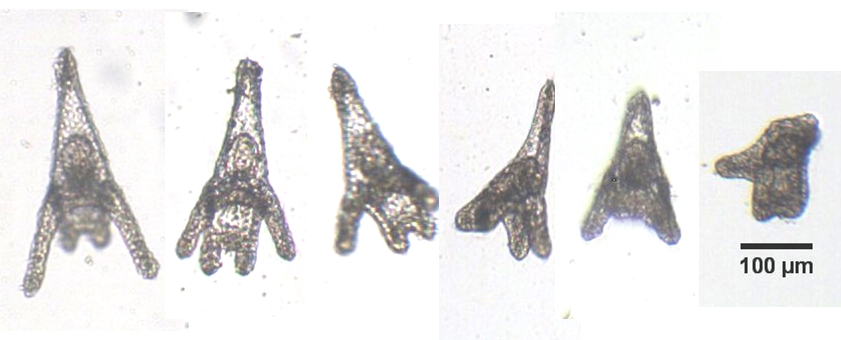


**Figure S1.** Measurement of maximum dimension in *P. lividus* larvae and embryos (A) and alterations in larval growth in *P. lividus* larvae resulting from exposed fertilized eggs.

**Table S1.** Summary of the parametric estimates and confidence intervals (α = 0.05) obtained by fitting Eq. (2) to the activity of glutathione S-transferase (GST) and glutathione reductase (GR) of sea urchin larva exposed to chlorpyrifos. Statistical values of adjusted coefficient of multiple determination (adj. R^2^) and p-values from Fisher’s F-test (α = 0.05) are also summarized.

| **Parameter** | **GST** | **GR** |
| --- | --- | --- |
|  |  |  |
| *Base* | 61.3±22.3 | 41.5±3.7 |
| *Amplitude* | 31.9±29.7 | 31.4±8.5 |
| *Mean* | 69.2±36.6 | 104.5±6.7 |
| *SD* | 44.1±42.6 | 40.2±14.6 |
| adj. R^2^ | 0.618 | 0.978 |
| p-value | 0.147 | 0.009 |
|  |  |  |

**Table S2**. Estimate of the parameters of the multiple linear regression models and its significance (0 ‘***’, 0.001 ‘**’, 0.01 ‘*’, 0.05 ‘.’, 0.1 ‘ ’, 1). Statistical values of adjusted coefficient of multiple determination (adj. R^2^) and p-values from Fisher’s F-test (α = 0.05) are also summarized.

| **Parameter** | **Chlorpyrifos** | **Triphenyl phosphate** | **Bisphenol A** |
| --- | --- | --- | --- |
|  |  |  |  |
| Intercept | 0.9197*** | 0.5627** | 1.2060*** |
| GST | 0.0004 | 0.0018 | -0.0010 |
| CAT | 0.0042 | -0.0459 | -0.0457* |
| GR | 0.0028* | 0.0002 | -0.0004 |
| AChE | -0.0014 | 0.0110** | 0.0018 |
| Concentration | -0.0013*** | -0.0004*** | 0.0000 |
| Adj. R^2^ | 0.9039 | 0.8914 | 0.2741 |
| p-value | 0.0013 | 0.0000 | 0.1120 |
|  |  |  |  |
